# Printable devices for neurotechnology

**DOI:** 10.3389/fnins.2024.1332827

**Published:** 2024-02-19

**Authors:** Rita Matta, David Moreau, Rodney O’Connor

**Affiliations:** ^1^Mines Saint-Etienne, Centre CMP, Departement BEL, Gardanne, France; ^2^Department of Chemical Engineering, Polytechnique Montreal, Montreal, QC, Canada

**Keywords:** printable devices, printable electronics, neurotechnology, microelectrode arrays, neuroprobes

## Abstract

Printable electronics for neurotechnology is a rapidly emerging field that leverages various printing techniques to fabricate electronic devices, offering advantages in rapid prototyping, scalability, and cost-effectiveness. These devices have promising applications in neurobiology, enabling the recording of neuronal signals and controlled drug delivery. This review provides an overview of printing techniques, materials used in neural device fabrication, and their applications. The printing techniques discussed include inkjet, screen printing, flexographic printing, 3D printing, and more. Each method has its unique advantages and challenges, ranging from precise printing and high resolution to material compatibility and scalability. Selecting the right materials for printable devices is crucial, considering factors like biocompatibility, flexibility, electrical properties, and durability. Conductive materials such as metallic nanoparticles and conducting polymers are commonly used in neurotechnology. Dielectric materials, like polyimide and polycaprolactone, play a vital role in device fabrication. Applications of printable devices in neurotechnology encompass various neuroprobes, electrocorticography arrays, and microelectrode arrays. These devices offer flexibility, biocompatibility, and scalability, making them cost-effective and suitable for preclinical research. However, several challenges need to be addressed, including biocompatibility, precision, electrical performance, long-term stability, and regulatory hurdles. This review highlights the potential of printable electronics in advancing our understanding of the brain and treating neurological disorders while emphasizing the importance of overcoming these challenges.

## 1 Introduction

Printable electronics for neurotechnology is a rapidly emerging field that involves the fabrication of electronic devices using printing techniques, such as inkjet printing (Adly et al., 2018; Garma et al., 2019; Borda et al., 2020), screen-printing (Yang et al., 2014), or 3D printing (Chen et al., 2020). Compared to traditional microelectronics, printable electronics offer advantages in terms of rapid prototyping, scalability, and cost-effectiveness, as they can be produced in large quantities using high-throughput printing techniques (Almasri et al., 2020). This makes them a potentially cost-effective solution for widespread adoption in clinical settings (Garma et al., 2019), whether it is for *in vitro* applications (Garma et al., 2019) or *in vivo* (Chen et al., 2020).

Printable neurotechnology devices are a promising alternative to traditional devices made from photolithographic processes. For example, printable electrodes can be used to record the electrical signals of neurons (Bachmann et al., 2017; Kokubo et al., 2019; Borda et al., 2020), while printable microfluidic channels can be used to deliver drugs or other substances to specific areas of the brain (Cui, 2023). The topic of organic and printed electronics is well-established in terms of academic, scientific, and technical research (Sommonte et al., 2023). Unlike traditional electronics, which typically rely on expensive and complex fabrication processes, printable electronics can be produced using low-cost, scalable methods that enable the creation of large-area, flexible, and lightweight devices. The use of printing techniques allows for the integration of different materials and components onto a single substrate, enabling the creation of complex, multi-functional devices. This makes them a potentially cost-effective solution for widespread adoption in clinical settings. One of the major advantages of printable electronics is their versatility and adaptability. It can be used to create a wide range of devices, from simple RFID tags to complex wearable electronics and biomedical sensors (Keskinen, 2012). It is also compatible with a variety of substrates, enabling the creation of devices that can conform to curved or irregular surfaces. Overall, printable devices for neuro-technology hold great promise for advancing our understanding of the brain and developing new treatments for neurological disorders. However, several challenges need to be addressed, including biocompatibility, durability, and performance, before these devices can be widely adopted for clinical use (Chen et al., 2018). This review describes the novel fabrication approaches related to printable devices for neuro-technology, evaluating their strengths and limitations from the perspective of end users to assist direct neuro-engineering efforts toward the demands of research endeavoring to interface with the brain.

## 2 Overview of printing techniques

The term “printed electronics” refers to the intersecting techniques of printing and electronics, and this innovative combination offers a more straightforward process for producing low-cost electronic components. Recently, there has been a surge in interest in adapting traditional printing processes to print novel functional materials, particularly using electronic inks for device fabrication. This approach allows one to directly print or manufacture interconnections, sensors, or microelectrode arrays for neuro-technology by exchanging traditional graphic inks for electrically functional inks.

Printing techniques can be separated into two categories: contact and non-contact printing. Roll-to-roll printing techniques are all contact methods except ink-jet printing, which is not traditional. Printing techniques usually involve transferring a pattern from a solid surface onto a substrate by physical contact, resulting in a two-dimensional design (Mathematics, 2016).

**Figure 1:**
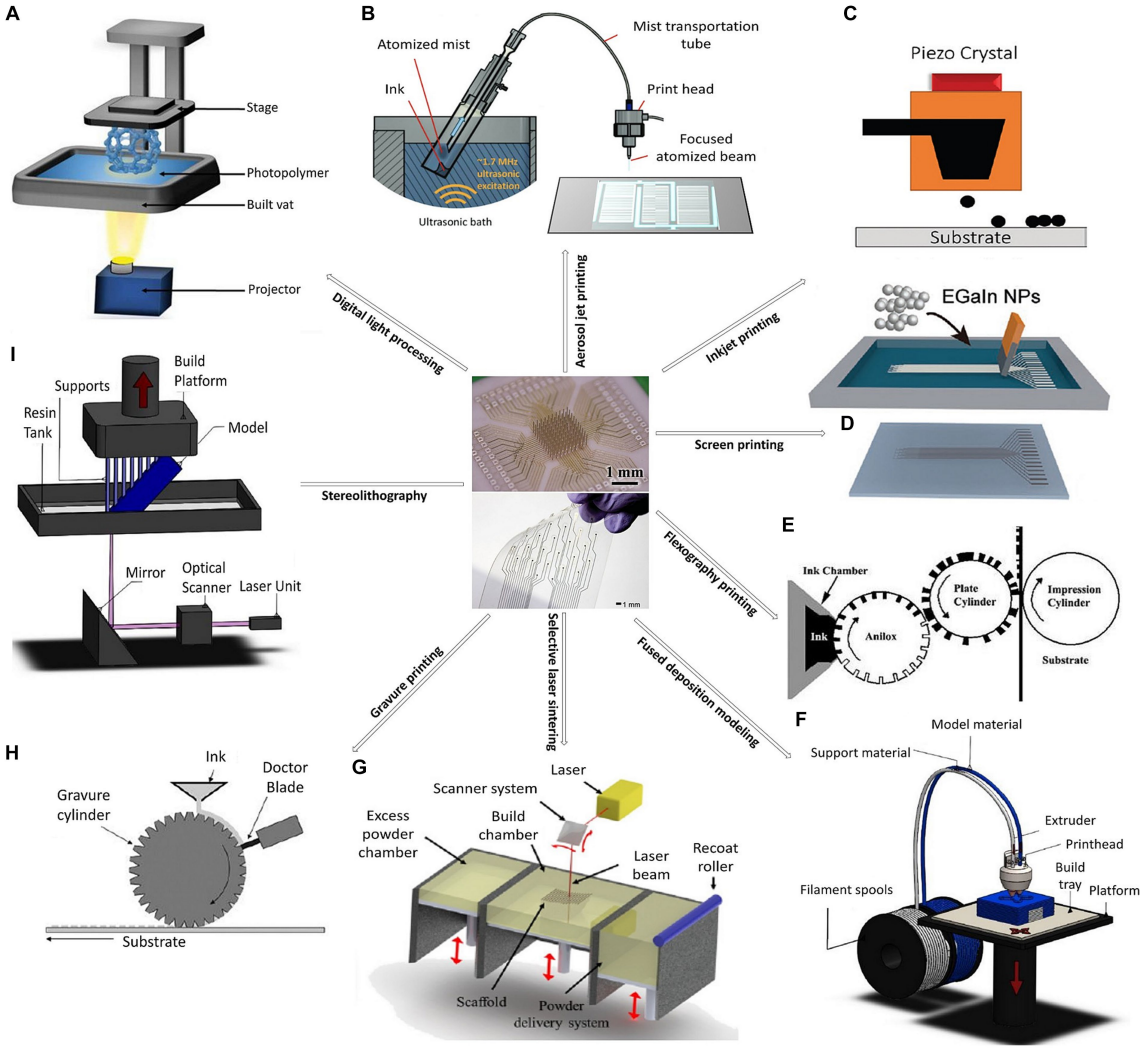
Fabrication of printable devices for neurotechnology, in the center respectively, a 3D nanoprinted microelectrode array platform, and inkjet-printed gold electrode arrays for bioelectronic interfaces (Reproduced from Khan et al. (2016) and Saleh et al. (2022)), employing: **(A)** Digital light processing (Reproduced from Market Business News (2023)). **(B)** Aerosol jet printing (Reproduced from Seiti et al. (2022)). **(C)** Inkjet printing (Reproduced from Cruz et al. (2018)). **(D)** Screen printing (Reproduced from Dong et al. (2021)). **(E)** Flexography printing (Reproduced from Khan et al. (2015)). **(F)** Fused deposition modeling (Reproduced from Szymczyk-Ziólkowska et al. (2020)), **(G)** Selective laser sintering (Reproduced from Puppi and Chiellini (2020)). **(H)** Gravure printing (Reproduced from Khan et al. (2015)). and **(I)** Stereolithography (Reproduced from Szymczyk-Ziólkowska et al. (2020)).

Unlike traditional fabrication methods, the process to make printable devices is less complicated and can be done in several ways (see Figure 1), including:

1. Inkjet printing: Inkjet printing is a drop-on-demand patterning process that uses micron precision to directly eject various types of functional materials. Micron precision indicates a high level of accuracy in terms of the size (diameter of the drop) and placement (positioning of ink droplets on the substrate). The combination of small droplet size and precise positioning will allow for high-resolution prints (10 μm). The conditions of wettability are influenced by several factors, like surface roughness, surface energy, temperature, surface treatment, etc. Some parameters that will also affect the quality of printing are the drop spacing, the waveform as well as the jetting frequency. All of these can be modified according to the nature of the substrate and the ink formulation to get the desired results and highest resolution possible (see Table 1). High-resolution prints of a variety of materials, including conductive inks and polymers, can be created using this non-contact and mask-less printing technique. Inkjet printing works by using a printhead that contains tiny nozzles or jets through which ink droplets are sprayed onto a surface, to make printable biosensors, drug delivery systems, and neural stimulation tools. Interest in the creation of electrodes for brain interfacing has recently increased due to inkjet printing (Derby, 2010; Bachmann et al., 2017; Adly et al., 2018; Garma et al., 2019; Kokubo et al., 2019; Almasri et al., 2020).
2. Screen printing: Screen printing techniques use a mesh stencil to transfer ink onto a substrate. With the use of a squeegee, ink is forced through the solid mesh’s open area during screen printing. For this type of printing, high-viscosity inks (see Table 1) with thixotropic (shear thinning) properties are necessary given that low-viscosity inks will simply flow through the screen due to gravity (Mathematics, 2016). While gravity can play a role, the primary driving forces are often the pressure applied to the squeegee and the rheological properties of the ink. More important key parameters that should be taken into consideration are the print speed and print gap (Borda et al., 2020). The resolution of screen printing depends on the mesh count of the screen and the properties of the ink. Higher mesh counts generally provide finer resolution. Moreover, the line width and gaps between features depend on the mesh size of the screen and the rheological properties of the ink. Without giving any consideration to proper tuning of the ink properties and mesh count, standard print resolutions fall within the range of 50-100 μm, with wet thicknesses measuring just a few microns (Khan et al., 2015). The two commonly used screen-printing techniques, flatbed and rotary screen printing, are suitable for roll-to-roll processing. The low-cost, high-throughput method of screen printing can be used to print conductive inks and polymers. It has been applied to the development of printable neural recording and stimulation devices (He et al., 2019).
3. Flexographic printing: Flexography printing is a roll-to-roll printing technique that, among other things, may imprint conductive inks, polymers, and biomolecules. This printing technique has been used to create sensors for wearable neurological monitoring. Flexography is a type of relief printing that makes use of flexible printing plates composed of photopolymer or rubber. The cylinder on which these plates are placed rotates while transferring ink to the printing substrate (Mathematics, 2016; Khan et al., 2019).
4. 3D printing: Additive technologies based on materials are technically referred to as 3D printing. This printing technique may create complex 3D structures by adding layers of material. Using 3D printing, devices for medication administration and neurological stimulation have been produced (Bagaria et al., 2018; Jakus, 2018).

- Aerosol jet printing: This method of non-contact printing uses a pneumatic atomizer to generate a fine ink aerosol that is then sprayed onto the substrate using a gas stream. The Aerosol jet technique may be used to print on a variety of materials, including conductive inks and adhesives, onto a substrate to create a 3D object or electronic devices (Agarwala et al., 2017; Seiti et al., 2022).
- Fused deposition modeling (FDM): A heated nozzle is used to melt and extrude thermoplastic filament layer by layer to create 3D geometries (Espalin et al., 2023).
- Digital light processing (DLP): DLP 3D printers use a digital light projector to project an entire layer of the object onto a vat of liquid resin. UV light, modulated by several millions of micromirrors, is used to solidify photocurable material layer by layer to form the object (Lopez-Larrea et al., 2022).
- Stereolithography (SLA): SLA printers use a laser to selectively solidify liquid resin. In stereolithography, the laser traces the shape of each layer to polymerize the photo-curable resin where it hits. The 3D shape is built layer by layer in this manner (Seck et al., 2010).
- Selective laser sintering (SLS): Laser is used to selectively fuse powdered material layer by layer to create 3D structures (Zhou et al., 2008).
- Gravure printing: In gravure printing, a cylinder with engraved protrusions is rotated over a moving substrate, such as paper or plastic. A doctor blade is used to scrape off excess ink from the cylinder’s protrusions, and the remaining ink, which has a low viscosity, is then transferred from the engraved cells to the substrate (Moonen et al., 2012; Mathematics, 2016).

## 3 Printable materials for neural device fabrication

Choosing the appropriate material for biological thin flexible neural implants is critical to ensure their safety, functionality, and longevity (see Table 1). Here are some main factors to consider when making this choice:

1. Biocompatibility: The material should not cause any adverse reactions or immune responses when implanted in the body. It should also be non-toxic and non-inflammatory (Ryynänen et al., 2023).
2. Flexibility: The implant should be flexible enough to conform to the curvature of the brain or other neural tissue without causing damage or discomfort (Almasri et al., 2020).
3. Electrical properties: The material should have suitable electrical properties for transmitting or receiving electrical signals from the neural tissue (Seker et al., 2010).
4. Durability: The material should be able to withstand the mechanical stresses and strains encountered during implantation and use (Borda et al., 2020).

**Table 1:** Overview of printing techniques, printable materials, resolution, viscosity, surface tension, advantages and disadvantages of each technique.

Printing technique	Materials	Resolution (μm)	Viscosity (cP or mPa.s)	Surface tension (mN/m)	Advantages	Disadvantages	Ref
Inkjet printing (2D)	Conductive inks (silver nanoparticles, gold nanoparticles), conducting polymers (PEDOT:PSS), PANI, PPy, biological materials, ceramics, etc.	10–100	1–25	20–60	-Precise printing, high resolution, fine patterns. -Low cost. -Does not require clean room facilities. -Precise control of droplet size and placement.	-Slow process compared to other printing techniques. -Challenging for complex electrode patterning. -Conductivity of the ink may be lower. -Limited material compatibility/ durability of the ink. -Coffee ring effect due to uneven distribution of dried materials.	Sirringhaus et al. (2000), Tobjörk and Österbacka (2011), Cummins and Desmulliez (2012), Yu et al. (2012), Khan et al. (2015), Kim et al. (2017), Garma et al. (2019), Shur et al. (2020), Lo et al. (2021), Benny Mattam et al. (2022), and Kim et al. (2022)
Gravure printing	PEDOT:PSS, PANI, PPy, PMMA, P105 (Butylene copolymer, Merck), F8T2 (ADS),etc.	50–200	50–200	30–50	-High resolution, good uniformity. -High printing speed. -Wide range of printable materials.	-High cost. -Slow production rate. -Defects-related challenges due to contact printing techniques.	Sirringhaus et al. (2000), Schmidt et al. (2010), Tobjörk and Österbacka (2011), Moonen et al. (2012), Khan et al. (2015), Mathematics (2016), Cruz et al. (2018), and Sico et al. (2018)
Flexography	PEDOT:PSS, PANi, PPy, copper particle ink, etc.	50	10–100	25–50	-Patterns are raised on a low-cost flexible plate. -Can be used with fragile and stiff substrates due to its high flexibility and minimal pressure applied to the substrate. -Improved pattern integrity and quality in both horizontal and vertical. -High throughput technique. -High resolution.	-Ink formulation must be controlled. -Marbling effect. -Layer cracks and non-uniform films.	Blayo and Pineaux (2005), Schmidt et al. (2010), Yu et al. (2012), Khan et al. (2015), and Mathematics (2016)
Screen printing	PEDOT:PSS, PANi, PPy, conductive inks (silver nanoparticles), etc.	10–500	500–10,000	35–50	-Low cost to produce large-area electrodes. -Rotary screen-printing gives a good edge definition. -The preferred approach for small-scale lab processes is flatbed screen printing. -Suitable for viscous inks. -No active material losses, during large-scale manufacturing.	-High viscosity required to prevent spreading. -Resolution can be lower compared to other printing techniques. -Rotary screen is more expensive than a flatbed and more difficult to clean. -Multilayer screen printing can be challenging in rotary. -Exposure of the ink to the atmosphere during printing.	Krebs et al. (2009), Alonso et al. (2010), Amb et al. (2012), Khan et al. (2015), Mathematics (2016), Shur et al. (2020), García-Miranda Ferrari et al. (2021), and Benny Mattam et al. (2022)
Aerosol jet printing	PEDOT:PSS, PANi, PPy, polymers, hydrogels, carbon-based solutions, nanoparticles inks, etc.	10	1–1,000	50–60	-Print high-resolution patterns. -Print on non-planar surfaces.	-Limited scalability and high cost of equipment. -Requires specialized equipment and expertise. -Distortion of very narrow shapes.	Agarwala et al. (2017), Gibney et al. (2017), Parate et al. (2020), Striani et al. (2021), Saleh et al. (2022), Seiti et al. (2022)
Extrusion printing/ Fused deposition modeling	PMMA, PCL, PLA, PCL-PU, collagen, gelatin, alginate, etc.	50–300	10^5^–10^8^	20–40	-Cost-effective and time efficient. -Offers design flexibility. -Can be used with a variety of materials suited for medical use.	-Limited resolution compared to other additive manufacturing techniques. -The physical properties of materials may influence the strength and durability of implants. -The heat could reduce cell viability if used for cellular applications.	Espalin et al. (2023), Khandan and Esmaeili (2019), Moroni et al. (2017), Sgrulletti et al. (2021), Ngo et al. (2018), Wang et al. (2017), Joung et al. (2020), and Lin et al. (2016)
Digital light processing	PEDOT_PEGDA, PCL, PLA, etc.	70–100	500–5,000	25–50	-High resolution. -Prints a continuous plane, which speeds up the printing process without losing resolution quality. -Suitable for complex geometries.	-Post-processing steps may be required. -Limited scalability for mass production.	Lopez-Larrea et al. (2022), Moroni et al. (2017), Ngo et al. (2018), Zhang et al. (2020), Shah et al. (2021), and Lebedevaite et al. (2021)
Stereolithography	Biomaterials, bio-inks, poly(ethylene glycol)/poly(D,L-lactide)-based resins, epoxy-based/ acrylate-based resins, etc.	10–70	600–5,000	30–60	-Very fine resolution. -Rapid fabrication that allows for rapid production of complex 3D shapes. -Design adaptability. -Compatible with various materials and cells. -Stereolithography is a nozzle-free printing technique.	-Expensive and slow process. - The use of specialized equipment such as stereo-lithography machines and lasers is required. -Only materials compatible with photo-crosslinking processes may be chosen. -Restricted to the photo-polymerization of a single spot while printing.	Seck et al. (2010), Arcaute et al. (2011), Moroni et al. (2017), Ngo et al. (2018), Wang et al. (2017), Joung et al. (2020), Gauvin et al. (2012), Sawan and Circuits (2015), Wang et al. (2015), and Ni et al. (2018)
Selective laser sintering	Thermoplastic polymers, PCL, polyvinyl alcohol, polyethylene glycol, polymethacrylate, etc.	100–400	–	–	-Control the porosity and mechanical properties. -Low cost. -Time efficient. -Minimal/ no post-processing steps. -Solvent-free method.	-Presence of polymeric grains. -Material limitation (only non-thermolabile). -Need for specialized equipment (laser sintering machine). -Limited resolution compared to other fabrication methods. -Process more complex than other printing techniques.	Zhou et al. (2008), Moroni et al. (2017), Tan et al. (2005), Minaev et al. (2020), Wu et al. (2008), and Charoo et al. (2020)

Finally, it is critical to consult with professionals in the area to choose the best material for a certain application. The material chosen will be determined by the precise requirements of the brain probe, as well as the manufacturing procedures and techniques available. Some important parameters that should also be taken into consideration are the chemical and rheological properties of the ink, as some printers are limited by these factors.

### 3.1 Conductive materials

The physical requirements of the printed pattern, such as conductivity, optical transparency, stability to bending, and adhesion are important considerations in making flexible electronics. The physicochemical properties of the ink, such as aggregation and stability, and its compatibility with the printing device all play a significant role in the selection of the conductive material (Kamyshny and Magdassi, 2014). While metals are commonly used as the primary conductive material in microelectrode array technology, there’s a growing exploration of non-metal alternatives, such as conductive polymers and oxides, offering benefits in flexibility and biocompatibility. These electrodes typically exhibit dimensions ranging from 10 to 100 μm, necessitating costly cleanroom microfabrication for their production (Garma et al., 2019). In addition, dispersed nanoparticles (NPs), a dissolved organometallic compound, or a conductive polymer (both dissolved and dispersed) can all be used as conductive materials (Singh et al., 2010; Pajor-Świerzy et al., 2022). These inks often contain additional constituents including dispersants, adhesion promoters, surfactants, thickeners, stabilizing agents, and other additives, regardless of the conductive material or solvent utilized (Cummins and Desmulliez, 2012).

#### 3.1.1 Metallic micro/nanoparticles

Biocompatible metals with outstanding electrical characteristics, such as gold, platinum, and titanium can be chosen as thin film metals. The synthesis of metal NPs, which is necessary to meet the demands for a certain performance of the devices to be printed, is a key step in the formulation of metal-based conductive inks. The average size and distribution, shape, and type of protective agents on the surface of the manufactured NPs are particularly crucial aspects. Carefully selecting the reaction conditions (temperature, pH, sequence, and rate of reagent addition), as well as the right functional additives can tune these characteristics. Due to their high conductivity (σ = 4.42 × 10^7^ S.m^−1^ for gold, and σ = 6.3 × 10^7^ S.m^−1^ for silver; Pajor-Świerzy et al., 2022) and low cost, metallic nanoparticles, in particular silver nanoparticles (Kimtan et al., 2014; Kim et al., 2022), and gold nanoparticles (Bachmann et al., 2017; Imai et al., 2023), are frequently employed in conductive inks for printed electronics (Jensen et al., 2011; Khan et al., 2016; Kim et al., 2017; Liu et al., 2019; Almasri et al., 2020), as well as platinum-based inks (Borda et al., 2020). The high surface area of these materials, however, can cause aggregation and poor ink stability, and they can be prone to oxidation and other stability issues (Kamyshny and Magdassi, 2014). Owing to their superior mechanical properties, as they tend to be stretchable, liquid metals were also used in screen printing, such as eutectic gallium–indium (Dong et al., 2021), as well as platinum/gold (Pt/Au) conductor paste (Duquesnoy et al., 2017).

Some parameters can influence the formation of the conductive layer when using nanoparticles. Nanoparticles have remarkable properties compared to bulk material. The smaller size and uniform dispersion of silver nanoparticles in inks make them suitable for printing applications. In inkjet printing, particles with larger size are not suitable for ink formation as they clog the nozzles (Nayak et al., 2019). Another important factor is the choice of solvent, and its evaporation rate, which can affect the dynamics of nanoparticles layers. The coffee ring effect, a common occurrence during the drying of nanoparticle-based inks, plays a role in the final deposition. As the solvent evaporates, nanoparticles migrate toward the outer edge of the droplet, resulting in a ring-like pattern, introducing non-uniformities in the printed layer. This effect can be reduced by using high concentration ink with a low evaporation rate carefully balancing these factors with the surface tension properties of the liquid (De Gans and Schubert, 2004). Higher nanoparticle concentration lead to a denser packing of nanoparticles affecting both the thickness and conductivity of the layer. Stabilizing agents, such as polyvinyl pyrrolidone (PVP), have been employed to prevent agglomeration, maintaining colloid stability of silver nanoparticles with diameter less than 50 nm (Wang et al., 2005). Surface treatment of the substrate is an additional consideration for optimizing the adhesion and performance of the conductive layer.

#### 3.1.2 Conductive polymers

Advances in conducting polymer technology have paved the path for all-polymer electronic components and circuits (De Gans et al., 2023). Conductive polymer coatings can improve the electrical performance of brain recording and stimulation electrodes by lowering the interfacial impedance and improving the charge transfer density (Benny Mattam et al., 2022). Because of their unique polymeric nature as well as favorable electrical and mechanical properties, stability, and biocompatibility, conducting polymers, a class of polymers with intrinsic electrical conductivity, have been one of the most promising materials in applications such as flexible electronics (Someya et al., 2016) and bioelectronics (Yuk et al., 2019). Polyaniline (PANi) (conductivity σ = 200 S.cm^−1^; Tobjörk and Österbacka, 2011), polypyrrole (PPy) (resistivity ρ = 1–1.5 × 10^6^ Ω.cm; Mabrook et al., 2006), and poly (3,4-ethylenedioxythiophene) (PEDOT) (conductivity σ = 1–155 S.cm^−1;^ Hill et al., 2023) are some of the most popular solution-processable conducting polymers utilized today (Tobjörk and Österbacka, 2011; Weng et al., 2014; Garma et al., 2019). Indeed, poly(3,4-ethylenedioxythiophene) doped with poly(styrene sulfonate) (PEDOT:PSS) has been extensively investigated in recent years for applications in bioelectronics due to their simplicity of manufacturing, mixed electronic/ionic conductivity, and biocompatibility (Bihar et al., 2017; Seiti et al., 2022). Only printing allows for the deposition of conducting polymers with spatial resolution in the x, and y planes in the tens of microns and layer thicknesses in the order of 100 nm (Weng et al., 2010; Yuk et al., 2023).

#### 3.1.3 Other conductive inks

Another advantage of printing techniques is the simplicity with which new ink materials, such as carbon nanotubes (CNTs) (conductivity σ = 103–104 S.cm^−1^; Tobjörk and Österbacka, 2011) or graphene, can be incorporated. Because of its electrochemical stability, and low impedance for electrical monitoring and stimulation of cellular activity, carbon nanoparticle ink was used to print the interface electrode material (Adly et al., 2018; Schnitker et al., 2018; Chen et al., 2020). On the other hand, inkjet-printed graphene ink can be used to fabricate flexible transparent films (Kamyshny and Magdassi, 2014). Moreover, graphene/PEDOT:PSS hybrid ink was also introduced to enhance the electrochemical performance of the electrodes and is comparable to 7 layers of printed PEDOT:PSS ink (Almasri et al., 2020).

### 3.2 Dielectric inks

Dielectric materials are electrical insulators that are frequently employed to separate conducting layers in electronic devices. Polyimide is a biocompatible, flexible polymer that has been utilized in several medical implants, including neurological probes. Polyimide-based (PI) ink was used as a backbone for neural interfaces, or a dielectric layer in so many applications (dielectric constant k = 3–4; Bachmann et al., 2017; Adly et al., 2018; Saleh et al., 2022; Liu et al., 2023). Another material that can serve as a dielectric layer is polycaprolactone (PCL), a bioresorbable polyester with outstanding mechanical properties (dielectric constant k = 3.2; Aguilar et al., 2012; Almasri et al., 2020). It was also shown that it is possible to print polyvinyl alcohol (PVA), a water-soluble synthetic polymer (dielectric constant k = 7.8; Kumar et al., 2014; Salaoru et al., 2016), and CYTOP fluoropolymer (dielectric constant k = 2; Schmidt et al., 2015; Khan et al., 2016; Lee et al., 2016; Li et al., 2016), as well as poly-4-vinylphenol (PVP) that was used to prepare a dielectric ink (dielectric constant k = 3.8; Kang et al., 2012; Kumar et al., 2014). SU-8, an epoxy-based photoresist, is another material of interest that is UV curable and can also be used as a passivation layer (dielectric constant k = 2.8; Bernasconi et al., 2019; Garma et al., 2019; Ghannam et al., 2023). A further example is PMMA (polymethylmethacrylate; dielectric constant k = 2.6–3,9; Kumar et al., 2014), an FDA-approved synthetic biomaterial that can be used in a variety of applications, including the fabrication of implants (Espalin et al., 2023). Moreover, dielectric prints were made of FDA nylon 680 resin co-polymer filament (Mirzaee and Noghanian, 2017). Polylactic acid (PLA), a biodegradable polymer that can be made from renewable resources (cornstarch) was also 3D printed. Even though, PLA (dielectric constant k = 1.7–2.8; Kuzmanić et al., 2023) can be an excellent electrical insulator, it can also be conductive by changing its physical properties when doped with micro or nanoparticles (Sun and Velásquez-García, 2017).

## 4 Applications of printable devices for neurotechnology

Multielectrode array (MEA) chips and probes are expensive to purchase commercially, ranging from a few hundred to thousands of euros. As a result, researchers often attempt to extend the lifetime or reuse these devices, which compromises the quality and reproducibility of acquired data. Repeated use of microelectrodes, particularly in biological applications, is known to degrade the electrodes and diminish signal quality while increasing the risk of cross-contamination due to the possibility of biological debris remaining on the devices after cleaning. Consequently, the development of printed technologies has the potential to dramatically enhance the field of neurotechnology for preclinical applications and 1 day could lead to improved patient outcomes for a variety of neurological conditions. These devices should endeavor to be flexible, minimally invasive, high-resolution, biocompatible, and scalable.

There have been several successful applications of printable devices for neurotechnology. For example, 3D printers have been used to fabricate minimally invasive microscale inorganic light-emitting diode-based neural probes to control neural circuit activity in freely behaving animals (Parker et al., 2022), as well as penetrating electrodes for neural cell stimulation (Chen et al., 2020; Saleh et al., 2022). Additionally, extracellular potentials from cardiac cell cultures were monitored, and high-quality electrophysiological signals were recorded using inkjet-printed microelectrode arrays (Garma et al., 2019), as well as gold-covered electrodes for bio-impedance and bio-potential measurements (Khan et al., 2016).

Electrocorticography (ECoG) is a traditional, invasive technique that uses an array of electrodes to capture the spatiotemporal summated neuronal signals from the cortical surface of the brain. *In vivo* tests were carried out by implanting flexible printed ECoG sensors in rats and effectively recording brain signals (Borda et al., 2020; Kim et al., 2022), as well as *in vitro* tests (Garma et al., 2019; Almasri et al., 2020). These all-printed electrocorticography arrays were demonstrated to not cause cytotoxic responses.

Printed devices are a more cost-effective fabrication process that would have a significant impact on research using microelectrode arrays, as it gives a wider distribution of devices, including laboratories with no access to clean room facilities. In addition, printing MEA devices make them affordable enough to be considered disposable, thus removing the problems associated with their reuse. In fact, 3D printed low-cost devices, used for neural stimulation in mice, were made for less than $1 USD, and were assembled in 17 min showing the cost effectiveness and utility of printing methods (Morrison et al., 2019). Printing devices also offer the potential for exploring and combining new technological strategies. For example, a microfluidic approach for fabricating graphene-based microelectrode arrays through inkjet printing enabled real-time monitoring of brain cells exposed to stress conditions (Niaraki et al., 2022). Overall, the results support the use of printed microelectrode arrays in neurological applications. Moreover, printers can enable device customization, accelerating the adoption of new technology beyond the laboratory.

Additive manufacturing is a versatile alternative to cleanroom processes for rapid and easy fabrication of 3D microelectrode arrays. For instance, 3D electrode arrays were printed using electro-hydrodynamic inkjet printing for extracellular recording of action potentials (Schnitker et al., 2018), employing commercial gold nanoparticles ink (Sadie and Subramanian, 2014), or PEDOT:PSS ink using aerosol jet printing for neural tissue engineering devices for electrically combined stimulations (Seiti et al., 2022). Another innovative 3D printing approach provided custom nerve repair technology tailored to anatomical geometries and enhanced with physical and biochemical cues that promote the regeneration of numerous neural pathways (Johnson et al., 2015). In addition, 3D printing can facilitate enhancing strategies for the regeneration of the long-range nerve guide by improving long-distance nerve guide regeneration strategies (Petcu et al., 2018). Moreover, another approach shows that the use of stereo-lithography with PEG poly(ethylene glycol) offers a promising prospect of rapid and effective production of complex 3D peripheral tissue-engineered scaffolds, as a nerve repair strategy(Arcaute et al., 2011).

An additional advantage of using printable devices is the fabrication of controlled drug release devices that would allow for spatiotemporal control of drug distribution, perhaps resolving some of the issues associated with systemic dosage. An illustrative example is the use of printed polypyrrole (PPy) microneedle arrays to control the release of dexamethasone for use in the nervous system (Huang, 2022).

## 5 Challenges and future directions

Developing and deploying of printable neurotechnology devices necessitates interdisciplinary collaboration across domains including materials science, electrical engineering, neuroscience, and biomedical engineering, as well as careful attention to the issues listed below:

1. Biocompatibility: One of the main challenges is ensuring that the printed devices are biocompatible with the surrounding neural tissue. The materials used in the printing process must be non-toxic and not induce an immune response or inflammation in the body (Almasri et al., 2020; Zaszczyńska et al., 2021). *In-vitro* biocompatibility has been assessed on some printed platinum/gold electrodes and it was shown that this material did not impair cell function (Duquesnoy et al., 2017). In addition, cytotoxicity tests were investigated on transparent PEDOT:PSS μECoG and cell viability was confirmed (Kimtan et al., 2014).
2. Resolution and precision: Printable devices for neurotechnology must have a high level of precision and resolution in order to accurately interface with the complex and delicate structures of the nervous system. This can be challenging when printing at the micro-and nano-scale, which is necessary for many neurotechnology applications. Indeed, for the moment, printable devices might not be able to achieve the resolution of microfabricated neuropixel devices (Steinmetz et al., 2018, 2021), however, the choice of a suitable printing technique can highly impact the output. For example, aerosol jet printing can achieve high resolution (up to 10 μm, see Table 1). Also, proper ink formulation could highly influence the devices’ overall performance (Khan et al., 2015; Cruz et al., 2018; Borda et al., 2020; Sommonte et al., 2023).
3. Electrical performance: The electrical performance of printable devices for neurotechnology is also critical, as the devices must be able to detect and stimulate neural activity with high fidelity. For example, the aim is to achieve low impedance values to enhance the quality of neural recordings [19.5kΩ at 1KHz for inkjet-printed PEDOT:PSS electrodes (Garma et al., 2019), 7.1kΩ at 1KHz for 3D printed carbon electrodes (Chen et al., 2020), 9.6KΩ at 1KHz for inkjet-printed platinum (Borda et al., 2020)]. In addition, having a high signal to noise ratio is essential for reliable signal reading. Another important parameter is charge storage capacity, which helps to assess the device’s ability to store and release electrical charges, as high charge storage capacity is often desirable to enable efficient and safe stimulation over extended periods of time. Furthermore, for safe and effective stimulation, while avoiding damage to the electrodes and surrounding tissues, charge injection limits should be found. Moreover, optimizing the printed materials’ conductivity, resistance, and other electrical properties is also required (Borda et al., 2020). Electrode impedance, charge storage capacity, and charge injection capacity measurements were performed on printed electrodes in various studies to assess the overall electrical performance of the devices with in some cases, successful proofs of concept of EcoG measurements and *in vivo* stimulation (Duquesnoy et al., 2017; Shur et al., 2020; Kim et al., 2022; Imai et al., 2023).
4. Post-printing conditions: Post-printing curing conditions are considered important factors for the overall performance of the device. Some inks need to be baked at low temperatures [130°C for polyimide ink (Schnitker et al., 2018)], and some need flash-annealing (Almasri et al., 2020), while some others require to be sintered at very high temperatures (≈1,300°C; Borda et al., 2020). In some cases, this process can limit the choice of substrate material (for example glass, thus having to compromise between sintering parameters and the performance of the ink; Charlot et al., 2015).
5. Long-term stability: Printable devices must be stable and durable over time, as they will be implanted or used for extended periods in the body. Ensuring the long-term stability of printed devices is therefore critical for their safety and efficacy (Parker et al., 2022; Espalin et al., 2023). Printed electrodes were continuously stimulated in PBS as well 100% fetal calf serum (FCS) for 21 days, and properties of the electrodes were compared before and after this intense stimulation, showing the stability of the devices (Duquesnoy et al., 2017). On another study, mechanical properties for durability have been assessed with the use of a bending test where no statistically significant change in line resistance was observed after 100.000 bending cycles up to bending radius of 9.1 mm (Borda et al., 2020).
6. Regulatory challenges: The development of printable devices for neurotechnology will also face regulatory challenges, as these devices will be subject to regulatory approval processes before they can be used in clinical practice. This requires demonstrating the printed devices’ safety, efficacy, and reliability through rigorous testing and validation. While printed devices for neurotechnology offer exciting possibilities for the fabrication, the material used should be thoroughly evaluated for biocompatibility, especially for implantable medical devices. This is important since there are only few approved materials for implants like medical-grade material such as polyetheretherketone (PEEK) (Honigmann et al., 2021).

## Author contributions

RM: Investigation, Validation, Writing – original draft, Writing – review & editing. DM: Supervision, Validation, Writing – review & editing. RO’C: Funding acquisition, Supervision, Validation, Writing – review & editing.
